# Hesperetin-Enhanced Metformin to Alleviate Cognitive Impairment via Gut–Brain Axis in Type 2 Diabetes Rats

**DOI:** 10.3390/ijms26051923

**Published:** 2025-02-23

**Authors:** Danyang Zhang, Xiaoshi He, Yinbo Wang, Xiaoyu Wang, Xiao Han, Haodong Liu, Yan Xing, Bo Jiang, Zhilong Xiu, Yongming Bao, Yuesheng Dong

**Affiliations:** 1 MOE Key Laboratory of Bio-Intelligent Manufacturing, School of Bioengineering, Dalian University of Technology, Dalian 116024, China; zhangdanyang96@mail.dlut.edu.cn (D.Z.); syingjjip@163.com (X.H.); 32147002@mail.dlut.edu.cn (X.W.); hanxiao981031@163.com (X.H.); liu18742060205@163.com (H.L.); docxyxy@163.com (Y.X.); bojiang@dlut.edu.cn (B.J.); zhlxiu@dlut.edu.cn (Z.X.); biosci@dlut.edu.cn (Y.B.); 2 Dianxi Research Institute, Dalian University of Technology, Baoshan 678000, China; dlutdx@dlut.edu.cn; 3 School of Ocean Science and Technology, Dalian University of Technology, Panjin 124221, China

**Keywords:** hesperetin, metformin, diabetic cognitive impairment, central insulin resistance, SCFAs, gut microbiota

## Abstract

Diabetes constitutes a risk factor for cognitive impairment, whereas insulin resistance serves as the shared pathogenesis underlying both diabetes and cognitive decline. The use of metformin for treating cognitive impairment remains controversial. The present study found that hesperetin, a flavanone derived from citrus peel, enhanced metformin’s efficacy in reducing blood sugar levels, improving insulin sensitivity, and ameliorating cognitive impairment in diabetic rats. Additionally, it reduced the required dosage of metformin to one-third of its conventional dose. Transcriptome analysis and 16S rRNA sequencing revealed that the activation of insulin and cyclic-adenosine monophosphate response element binding protein (CREB)/brain-derived neurotrophic factor (BDNF) pathways benefited from the regulation of gut microbiota and the promotion of short-chain fatty acid (SCFA) producers such as *Romboutsia*. Furthermore, this study demonstrated that hesperetin supplementation counteracted the upregulation of β-site amyloid precursor protein cleaving enzyme 1 (BACE1), a pathological factor of Alzheimer’s disease (AD) that was induced by metformin. Our findings reveal that hesperetin can be used in supplementary treatment for cognitive impairment associated with diabetes.

## 1. Introduction

Type 2 diabetes mellitus (T2DM) is a prevalent systemic metabolic disorder, affecting over 483 million individuals worldwide in 2021 [1]. It is characterized by hyperglycemia due to insulin resistance [2] and has been associated with cognitive dysfunction accompanied by memory loss and cognitive decline, which poses challenges for effective reversal strategies. A large cohort study found that individuals that suffered from T2DM exhibited a twofold increased risk of subsequent dementia in comparison to those without T2DM [3]. Therefore, there is an urgent need to explore efficacious intervention strategies for diabetic cognitive impairment.

Insulin resistance, a primary etiology of T2DM, disrupts glucose metabolism and is considered the underlying mechanism for cognitive decline induced by diabetes in relation to central insulin resistance within the brain [4]. Given the pathophysiological commonalities, traditional euglycemic agents, such as metformin (N, N-dimethylbiguanide), show potential for ameliorating cognitive decline [5,6]. In particular, this agent has been reported to reduce the risk of cognitive impairment in diabetic patients [7]. Nevertheless, the role of metformin in the central nervous system remains controversial. It has been shown that metformin has no significant effect on improving cognitive function [8]. More importantly, a recent study demonstrated that long-term metformin treatment impaired cognitive function in AD mice and also had a negative effect on the amyloidogenic pathway in db/db mice [9]. A prospective study in individuals with T2DM also indicated that the use of metformin was associated with an elevated risk of mild cognitive impairment [10]. Furthermore, metformin could regulate the gut microbiota [11]. However, as the main side effect of metformin, some of the patients experienced gastrointestinal symptoms in a pilot randomized placebo-controlled clinical trial evaluating the impact of metformin on amnestic mild cognitive impairment [12]. This trial revealed limitations inherent to monotherapy and emphasized the need to consider alternative interventions rather than relying solely on one drug.

Drug combinations have been reported to enhance the management of diabetes and its associated complications while reducing side effects and improving efficacy with multiple targets. It has previously been demonstrated that baicalein and acarbose improved nonalcoholic fatty liver disease associated with prediabetes [13]. Therefore, combination therapy containing natural drugs holds promise for the management of diabetic complications.

Dietary flavonoids have a gentle mode of action and possess a high level of safety, along with diverse biological activities. Our group demonstrated that baicalin could serve as an adjunctive therapy to potentiate metformin’s insulin-sensitizing effects for T2DM prevention through microenvironment modulation [14]. Hesperetin, another citrus-derived dietary flavanone, exhibits health-promoting effects through both anti-diabetes [15] and neuroprotective activities [16]. Notably, our recent in vitro experiments revealed that hesperetin and metformin exerted a synergistic effect on protecting PC-12 neuroblastoma cells against methylglyoxal-induced damage [17].

The gut microbiota has a significant impact on cognitive decline via the gut–brain axis, where short-chain fatty acids (SCFAs) serve as a crucial medium in bidirectional gut–brain communication. Notably, butyrate has been reported to improve memory and reduce amyloid beta levels in AD mice [18]. Furthermore, valeric acid has been reported to inhibit amyloid-β_42_ (Aβ_42_) dose-dependently, which is beneficial for AD patients [19]. In addition, diabetes induced the disruption of gut microbiota and abnormal levels of SCFAs [20]. The gut microbiota may serve as a potential link between diabetes and cognitive impairment. Multiple studies have consistently demonstrated that flavonoids regulate the composition of the gut microbiota, exerting an influence on brain health [21]. A hesperetin diet was also proven to regulate the composition of the gut microbiota and the levels of SCFAs in rats [22]. Therefore, the gut microbiota could be a promising therapeutic target for diabetes-induced cognitive impairment.

Collectively, the current evidence indicates that the efficacy of the combination therapy of hesperetin and metformin in ameliorating diabetic cognitive impairment in vivo and its underlying mechanisms remains unexplored. To address this gap, the present study was designed to systematically evaluate the synergistic neuroprotective effects of hesperetin–metformin combination therapy in high-fat-diet- and streptozotocin (STZ)-induced T2DM rats and to elucidate potential mechanisms. This investigation aims to provide a combination therapy strategy to assist in managing cognitive impairment associated with T2DM.

## 2. Results

A high-fat diet combined with STZ injection is a common method for establishing rodent models of T2DM, and in recent years, it has also been used for research on diabetes-related cognitive impairment [23]. The rodent in this model exhibited significant memory decline and nerve damage, making it a reliable model for studying diabetes-related cognitive impairment [24]. In this study, as expected, cognitive impairment was successfully observed in an in vivo T2DM rat model after a six-week intervention period, enabling the comparison and analysis of the effects of the combination of hesperetin and metformin on glucose metabolism, insulin levels, and cognitive abilities in diabetic rats.

### 2.1. Effect of Combination of Hesperetin and Metformin on Glucose Metabolism in DM Rats

Initially, the effect on body weight was recorded weekly throughout the study period (Figure S1). Compared with the normal diet control (NC) group, the diabetes mellitus (DM) group exhibited a significant reduction in body weight following STZ administration. Furthermore, the low dose metformin+hesperetin (LM+HT) group, which received hesperetin supplementation at one-third of the conventional metformin dosage, exhibited a remarkable improvement in weight gain compared with the DM group.

**Figure 1:**
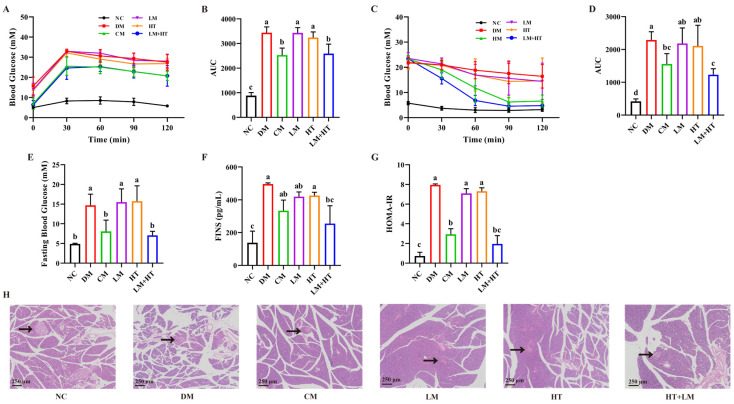
Effect of combination of hesperetin and metformin on glucose homeostasis and insulin resistance in diabetes mellitus (DM) rats. (**A**) Oral glucose tolerance test (OGTT) curve. (**B**) Area under the curve (AUC) of OGTT. (**C**) Insulin tolerance test (ITT) curve. (**D**) AUC of ITT. (**E**) Fasting blood glucose (FBG). (**F**) Fasting insulin (FINS) in serum. (**G**) Homeostatic model assessment for insulin resistance (HOMA-IR). (**H**) Hematoxylin–eosin (H&E) staining of pancreas. Black arrow indicated pancreatic islet. Scale bar = 250 μm. (*n* = 8) Data were presented as the mean ± SD. Groups sharing the same letter are not significantly different from each other, whereas groups with different letters exhibit statistically significant differences (*p* < 0.05). NC, normal diet control group. DM, diabetes mellitus group. CM, conventional-dose metformin (300 mg·kg^−1^·day^−1^) group. LM, low-dose metformin (100 mg·kg^−1^·day^−1^) group. HT, hesperetin (50 mg·kg^−1^·day^−1^) group. LM+HT, low-dose metformin + hesperetin (100 mg·kg^−1^·day^−1^ metformin + 50 mg·kg^−1^·day^−1^ hesperetin).

The primary objective of complication management is to ensure optimal blood glucose control. An oral glucose tolerance test (OGTT) was performed after a six-week intervention period to monitor blood glucose levels. As shown in Figure 1A,B, OGTT was impaired in diabetic rats (DM group) within six weeks. The administration of either a low dose of metformin (LM group) or hesperetin (HT group) alone did not significantly reduce blood glucose levels compared with the DM group (*p* > 0.05). Meanwhile, LM+HT reduced blood glucose significantly compared to the DM group (*p* < 0.05), which was comparable to the effect of a conventional dose of metformin (CM group) used alone. Additionally, an intervention with LM+HT in DM rats led to a significant decrease in GSP (glycosylated serum protein) levels compared with the DM group (*p* < 0.05) (Figure S2), indicating the temporary stabilization of blood glucose at a lower level.

### 2.2. Effect of Hesperetin Supplementation with Metformin on the Insulin Resistance in DM Rats

Similar results were observed in insulin tolerance tests (ITTs) (Figure 1C,D). Following a 120 min insulin injection, a slight decline in blood glucose was observed in the DM group, indicating a reduction in insulin sensitivity. The area under the curve (AUC) was increased in the DM group significantly compared with the NC group (*p* < 0.05). Neither the LM nor HT group exhibited any significant reduction in the AUC during the ITT. However, the LM+HT group demonstrated a significant decrease in the AUC compared with the DM group (*p* < 0.05). Similar results were found for fasting insulin (FINS) (Figure 1F), which was elevated in the DM group. The administration of LM+HT reduced the FINS significantly compared with the DM group (*p* < 0.05). The homeostatic model assessment for insulin resistance (HOMA-IR) index (Figure 1G) was calculated according to the fasting blood glucose (FBG) (Figure 1E) and FINS, revealing that hesperetin enhanced metformin’s effect on insulin sensitivity in rats with DM. The pancreatic islet morphology was further investigated via hematoxylin–eosin (H&E) staining. As illustrated in Figure 1H, pancreatic islets from NC rats displayed distinct boundaries, while those from DM rats exhibited evident atrophy and an irregular shape. Neither the LM nor the HT intervention had a significant impact on pancreatic islet morphology. However, LM+HT effectively restored pancreatic islet atrophy to a level similar to that seen in CM-treated rats. Therefore, supplementation with hesperetin was found to enhance metformin’s efficacy in ameliorating glucose metabolism and alleviating insulin resistance associated with pancreatic islet function impairment in diabetic rats.

### 2.3. Effect of Combination of Hesperetin and Metformin on Cognitive Impairment in DM Rats

**Figure 2:**
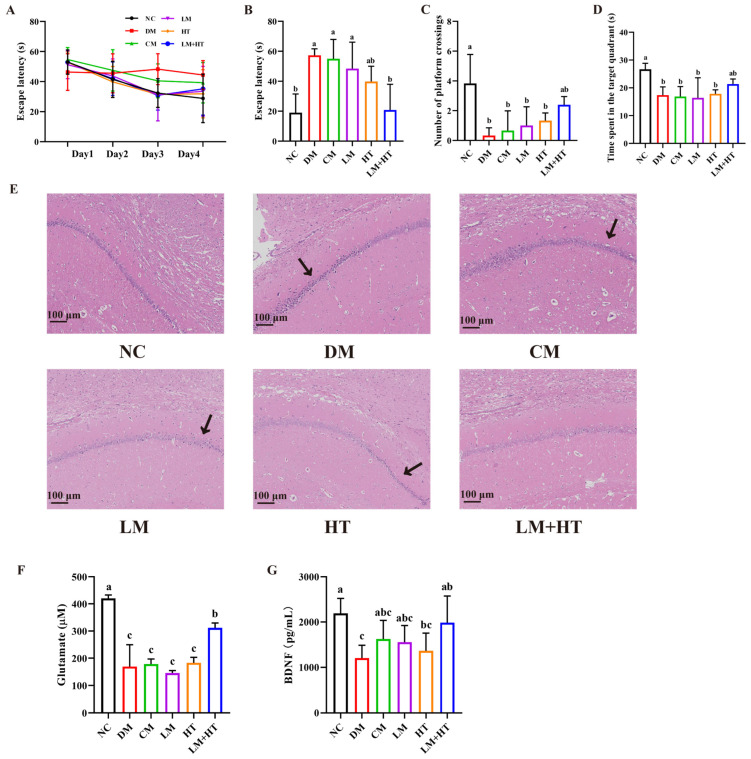
Effect of combination of hesperetin and metformin on Morris water maze (MWM) test of neuron damage in diabetes mellitus (DM) rats. (**A**) Escape latency during the training days. (**B**) Escape latency in probe test. (**C**) Number of platform crossings. (**D**) Time spent in the quadrant. (**E**) Hematoxylin–eosin (H&E) staining of hippocampus. Black arrow indicated hippocampal neuronal cells. (**F**) Glutamate in serum. (**G**) Brain-derived neurotrophic factor (BDNF) in serum. (*n* = 8). Data were presented as the mean ± SD. Groups sharing the same letter are not significantly different from each other, whereas groups with different letters exhibit statistically significant differences (*p* < 0.05). NC, normal diet control group. DM, diabetes mellitus group. CM, conventional-dose metformin (300 mg·kg^−1^·day^−1^) group. LM, low-dose metformin (100 mg·kg^−1^·day^−1^) group. HT, hesperetin (50 mg·kg^−1^·day^−1^) group. LM+HT, low-dose metformin + hesperetin (100 mg·kg^−1^·day^−1^ metformin + 50 mg·kg^−1^·day^−1^ hesperetin). DG, dentate gyrus.

The cognitive dysfunction induced by diabetes was assessed using the Morris water maze (MWM) test to investigate the effects of the combination of hesperetin and metformin. The results demonstrated that the DM rats took longer to reach the platform starting from day 3 compared with the rats in the NC group. However, the administration of LM+HT significantly reduced the escape latencies, indicating that the impaired spatial learning ability in DM rats was reversed by LM+HT (Figure 2A). Following the completion of training sessions, probe trial tests were conducted. The removal of the escape platform revealed significant memory impairment in DM rats, as evidenced by their increased latency in locating the platform, the decreased time spent in the target quadrant, and the number of crossings of the platform compared with the NC group (*p* < 0.05). Only LM+HT exhibited a significant increase in escape latency while restoring the time spent in the target quadrant and crossing target numbers to levels comparable to those observed in the NC group (*p* > 0.05) (Figure 2B–D). The single administration of CM, LM, and HT had no effect on the DM rats in the MWM test (*p* > 0.05). These findings support the involvement of a combination of hesperetin and metformin in restoring diabetes-induced cognitive dysfunction.

### 2.4. Effect of Combination of Hesperetin and Metformin on Neuronal Injury in the Hippocampus in DM Rats

The neuroprotective effect of LM+HT was investigated by examining histopathological changes using H&E staining and Nissl staining. As illustrated in Figure 2E, the DM group exhibited significant alterations in the morphology of hippocampal neuronal cells compared with the NC group. Nuclear pyknosis and neuronal damage were observed in the DM rats. However, the intervention with LM+HT restored neuronal damage more effectively than hesperetin alone. Notably, the conventional-dose metformin treatment had no effect on neuron damage in the DM rats, which was consistent with behavioral test results. The results of the Nissl staining are presented in Figure S3. The neurons’ nuclei in the NC group were round and dark-colored, and the cytoplasm was light-colored. The Nissl substance was damaged in the DM group and out of shape. LM+HT administration improved this damage in the DM rats. Furthermore, both serum glutamate (Figure 2F) and BDNF (Figure 2G) decreased significantly in the DM rats compared with the NC group (*p* < 0.05). No significant differences were observed between the CM, LM, HT groups, and the DM group (*p* > 0.05). Only the LM+HT group improved the content significantly compared with the DM group (*p* < 0.05).

### 2.5. The Mechanism of the Combination of Hesperetin and Metformin in the Hippocampus in DM Rats

**Figure 3:**
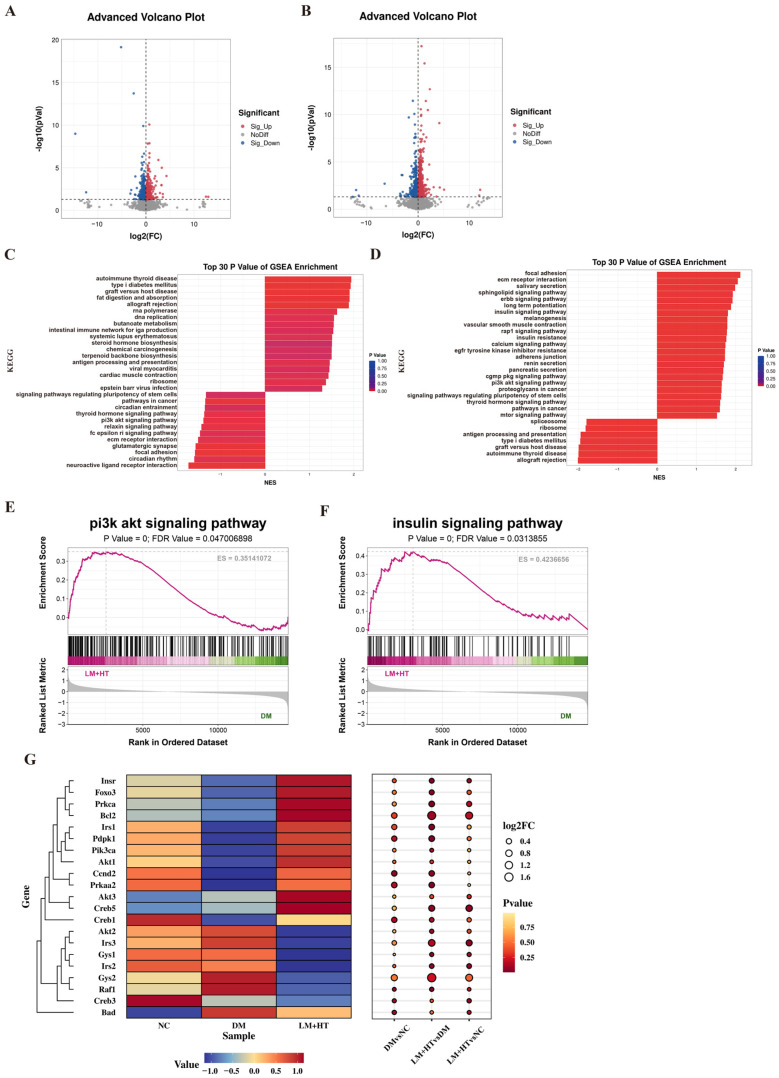
Effect of combination of hesperetin and metformin on transcriptome sequencing. (**A**) Volcano plot of DM vs. NC. (**B**) Volcano plot of HT+LM vs. DM. (**C**) Gene set enrichment analysis (GSEA); enrichment of DM vs. NC. (**D**) GSEA enrichment of HT+LM vs. DM. (**E**) GSEA of PI3K/AKT signaling pathway of HT+LM vs. DM group. (**F**) GSEA of insulin signaling pathway of HT+LM vs. DM group. (**G**) Heat map and bubble map of PI3K/AKT signaling pathway. (*n* = 3). NC, normal diet control group. DM, diabetes mellitus group. LM+HT, low-dose metformin+hesperetin (100 mg·kg^−1^·day^−1^ metformin + 50 mg·kg^−1^·day^−1^ hesperetin).

To identify genes associated with DM-induced rats in the hippocampus, genome transcriptomic analysis on the three experimental groups was performed. Volcano plots are presented in Figure 3. In comparison to the NC group, 248 genes were upregulated, and 185 genes were downregulated in the DM group (Figure 3A). Similarly, when comparing the LM+HT group to the DM group, 271 genes were upregulated and 215 genes were downregulated (Figure 3B). The enrichment in the Kyoto encyclopedia of genes and genomes (KEGG) and heat maps of differentially expressed genes in various groups are shown in Figures S4 and S5. Moreover, gene set enrichment analysis (GSEA) was conducted for the KEGG pathway enrichment analysis. Notably, significant enrichments were observed for the “phosphatidylinositol-3 kinase (PI3K)/ protein kinase B (AKT) signaling pathway,” “Insulin resistance,” and “Insulin signaling pathway” between DM vs. NC, as illustrated in Figure 3C, while LM+HT vs. DM comparisons showed enrichments for these pathways, as demonstrated in Figure 3D. The GSEA enrichment plots for the PI3K/AKT signaling pathway and insulin resistance are shown in Figure 3E and Figure 3F, respectively (*p* < 0.05, FDR < 0.25). Additionally, a heat map along with a bubble map illustrating gene expression patterns within the PI3K/AKT signaling pathway is presented in Figure 3G.

**Figure 4:**
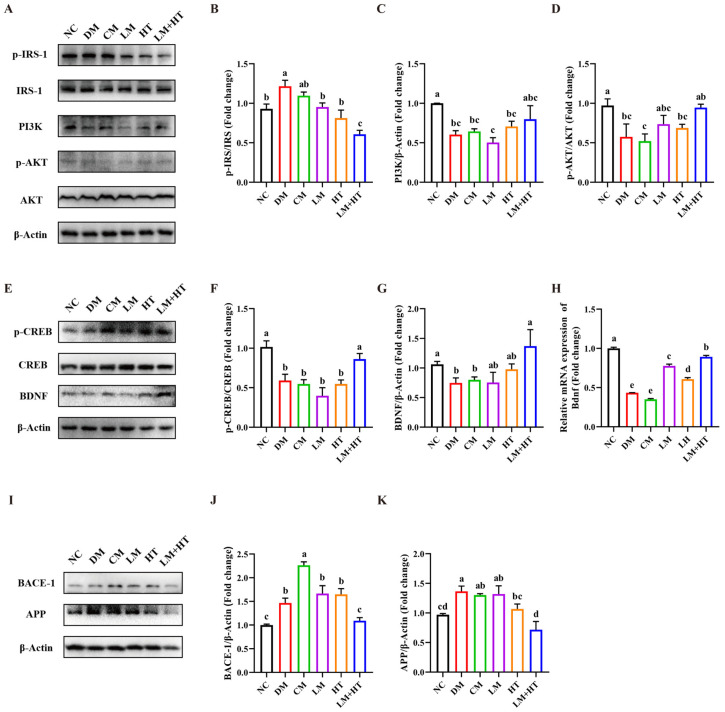
Effect of combination of hesperetin and metformin on insulin and CREB/BDNF pathways. Western blotting (**A**) and quantitative analysis of p-IRS-1/IRS-1 (**B**), phosphoinositide 3-kinase (PI3K)/protein kinase B (**C**), and p-AKT/AKT (**D**) in hippocampus. Western blotting (**E**) and quantitative analysis of p-CREB/CREB (**F**) and BDNF (**H**) in hippocampus. (**G**) *Bdnf* mRNA expression in hippocampus. Western blotting (**I**) and quantitative analysis of BACE-1 (**J**) and APP (**K**) in hippocampus. Data are presented as the mean ± SD. Groups sharing the same letter are not significantly different from each other, whereas groups with different letters exhibit statistically significant differences (*p* < 0.05). NC, normal diet control group. DM, diabetes mellitus group. CM, conventional-dose metformin (300 mg·kg^−1^·day^−1^) group. LM, low-dose metformin (100 mg·kg^−1^·day^−1^) group. HT, hesperetin (50 mg·kg^−1^·day^−1^) group. LM+HT, low-dose metformin + hesperetin (100 mg·kg^−1^·day^−1^ metformin + 50 mg·kg^−1^·day^−1^ hesperetin).

To confirm the underlying mechanisms through which the combination of hesperetin and metformin ameliorated diabetes-induced cognitive impairment, alterations in the insulin receptor substrates (IRS)/AKT/CREB/BDNF pathway in the hippocampus were detected using Western blot. As shown in Figure 4, there was a significant increase in the protein expression levels of p-AKT, p-IRS, and PI3K in the DM groups compared with the NC group (*p* < 0.05). The administration of either metformin or hesperetin alone did not exert any effect on the expression of these proteins (*p* > 0.05). The expressions of p-AKT and PI3K were upregulated, while the expression of p-IRS was downregulated significantly by administration for the LM+HT group compared with the DM group (*p* < 0.05). Concurrently, CREB activation occurred, as evidenced by the enhanced phosphorylation upon administration for the LM+HT group relative to the DM group (*p* < 0.05), consequently leading to a substantial upregulation of the downstream target protein BDNF compared with the DM group (*p* < 0.05).

Furthermore, the expressions of BACE-1 and amyloid precursor protein (APP) were detected. In comparison with the DM group, the CM group exhibited a significantly higher expression of BACE-1 (*p* < 0.05), while no significant differences were observed in the LM and HT groups (*p* > 0.05). However, there was a significant reduction in BACE-1 expression in the LM+HT group compared with the DM group. Similarly, when comparing APP expression in the DM group, both the CM and LM groups did not show any significant changes (*p* > 0.05). Both the HT and LM+HT groups reduced the expression of APP, with a further significant reduction observed in the LM+HT group compared with the HT group (*p* < 0.05). These results indicated that the combination of hesperetin and metformin downregulated BACE-1 and APP expressions.

### 2.6. The Combination of Hesperetin and Metformin Modulated the Gut Microbiota

**Figure 5:**
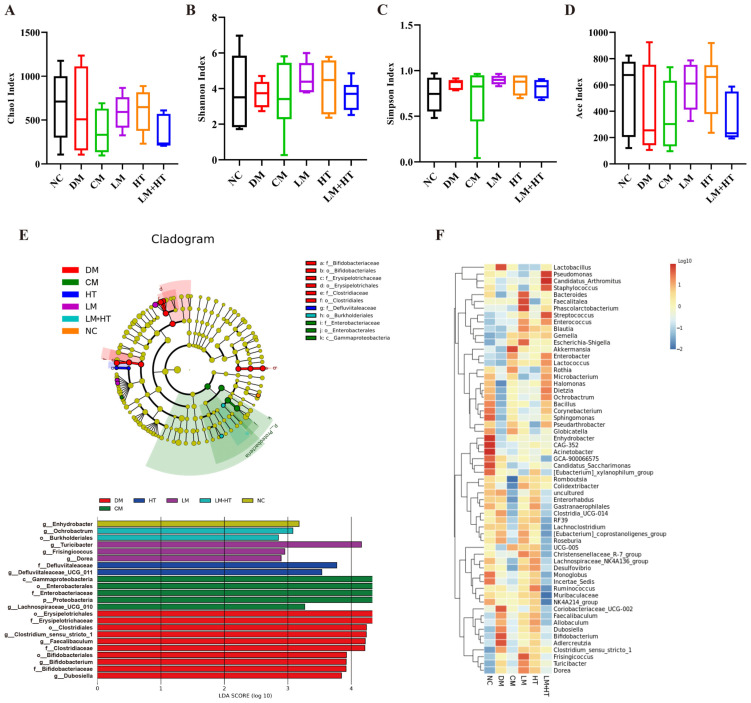
Effect of combination of hesperetin and metformin on gut microbiota. Alpha diversity index analysis. (**A**) Chao 1, (**B**) Shannon, (**C**) Simpson, and (**D**) ACE. (**E**) Cladogram generated from linear discriminant analysis (LDA) of effect size (Lefse). (**F**) Heat map. (*n* = 5). NC, normal diet control group. DM, diabetes mellitus group. CM, conventional-dose metformin (300 mg·kg^−1^·day^−1^) group. LM, low-dose metformin (100 mg·kg^−1^·day^−1^) group. HT, hesperetin (50 mg·kg^−1^·day^−1^) group. LM+HT, low-dose metformin + hesperetin (100 mg·kg^−1^·day^−1^ metformin + 50 mg·kg^−1^·day^−1^ hesperetin).

Recently, a growing amount of the literature has highlighted the crucial role of gut microbiota in cognitive function, emphasizing that their significance should not be overlooked [25]. To further investigate the underlying mechanisms behind the effects of the supplementation of hesperetin with metformin in DM rats, 16S rRNA microbiota analysis was conducted on fecal samples from rats. The α-diversity indexes (Figure 5), including the Chao1 (Figure 5A), Shannon (Figure 5B), Simpson (Figure 5C) and Ace (Figure 5D) indexes, showed no significant differences (*p* > 0.05).

Subsequently, linear discriminant analysis of effect size (LEfSe) based on linear discriminant analysis (LDA) was employed to compare the high-dimensional categories and identify the dominant bacterial communities among the various groups (Figure 5E). The corresponding heatmap is presented in Figure 5F. The results revealed that *Erysipelotrichaceae* (from the order *Erysipelotrichales* to the genus) played a pivotal role in inducing intestinal microbiota imbalance in the DM group. *Ochrobactrum* (from the order *Burkholderiales* to the genus) emerged as a key bacterial type in the LM+HT group. At the genus level, *Enhydrobacter*, *Turicibacter*, and *Defluviitaleaceae_UCG_011* were identified as key bacterial types in the NC, LM, and HT groups, respectively.

**Figure 6:**
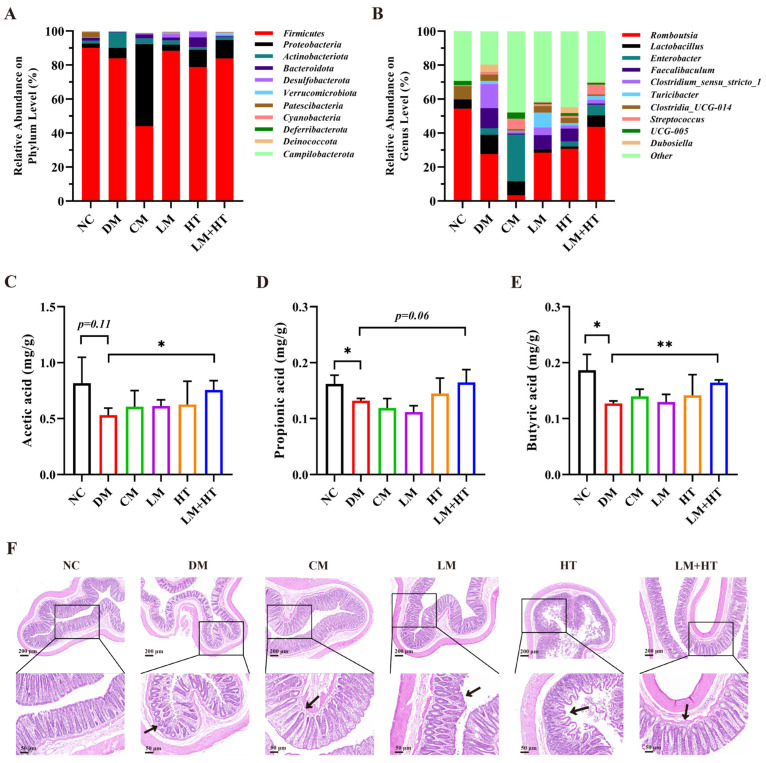
Effect of combination of hesperetin and metformin on relative abundance and SCFAs. Relative abundance at phylum level (**A**) and genus level (**B**). Relative abundance of *Firmicutes*/*Bacteroidetes* (**C**) The content of acetic acid in the colon. (**D**) The content of propionic acid in the colon. (**E**) The content of butyric acid in the colon. (**F**) Hematoxylin–eosin (H&E) staining of colon. Black arrow indicated crypt. Data are presented as the mean ± SD. * *p* < 0.05, ** *p* < 0.01. NC, normal diet control group. DM, diabetes mellitus group. CM, conventional-dose metformin (300 mg·kg^−1^·day^−1^) group. LM, low-dose metformin (100 mg·kg^−1^·day^−1^) group. HT, hesperetin (50 mg·kg^−1^·day^−1^) group. LM+HT, low-dose metformin + hesperetin (100 mg·kg^−1^·day^−1^ metformin + 50 mg·kg^–1^·day^−1^ hesperetin).

At the phylum level (Figure 6A and Figure S6A), the 11 phyla with the highest relative abundance of gut microbiota in rats were selected. Among them, *Firmicutes* and *Bacteroides* were the dominant bacteria, accounting for approximately 90% of the intestinal microbiota. At the phylum level, *Proteobacteria* were increased in the CM group but were significantly reduced in the LM+HT group (*p* < 0.05) The top 10 genera in relative abundance in each group are presented in Figure 6B and Figure S6B. Notably, *Romboutsia*, *Lactobacillus*, and *Clostridia_UCG-014* were found to be the dominant bacterial genera of the NC group. Conversely, *Romboutsia*, *Lactobacillus*, *Faecalibaculum*, and *Clostridum_sensu_stricto_1* constituted major components of the DM group’s microbiotic composition. In contrast, the LM+HT group was mainly composed of *Romboutsia* and *Lactobacillus*. As shown in Figure S7, compared with the NC group, the relative abundance of *Romboutsia*, *Halomonas* and *Orchrobactrum* were markedly decreased in the DM group, while *Faecalibaculum*, *Clostridia_UCG-014*, *Bifidobacterium*, *Clostridum_sensu_stricto_1*, *Desulfovibrio*, and *Muribaculaceae* obviously increased, which was reversed to varying degrees though the gavage administration of LM+HT. In brief, the combination of hesperetin and metformin could reverse the changes in the composition of intestinal microbiota induced by DM.

### 2.7. Regulation of IRS/AKT/CREB/BDNF Pathway Through SCFA Secreted by Gut Microbiota

As shown in Figure 6C–E, the SCFAs were determined. The concentrations of the SCFAs in the DM group were significantly decreased compared with those in the NC group (*p* < 0.05). Meanwhile, the concentrations of acetic acid, propionic acid, and butyric acid were significantly improved in the LM+HT group compared with that in the DM group (*p* < 0.05).

The colon morphology was assessed using H&E staining (Figure 6F). It was observed that the colon of rats in the NC group exhibited a distinct and well-defined crypt structure. In contrast, DM rats displayed evident crypt deformities, which were ameliorated upon intervention with LM+HT.

**Figure 7:**
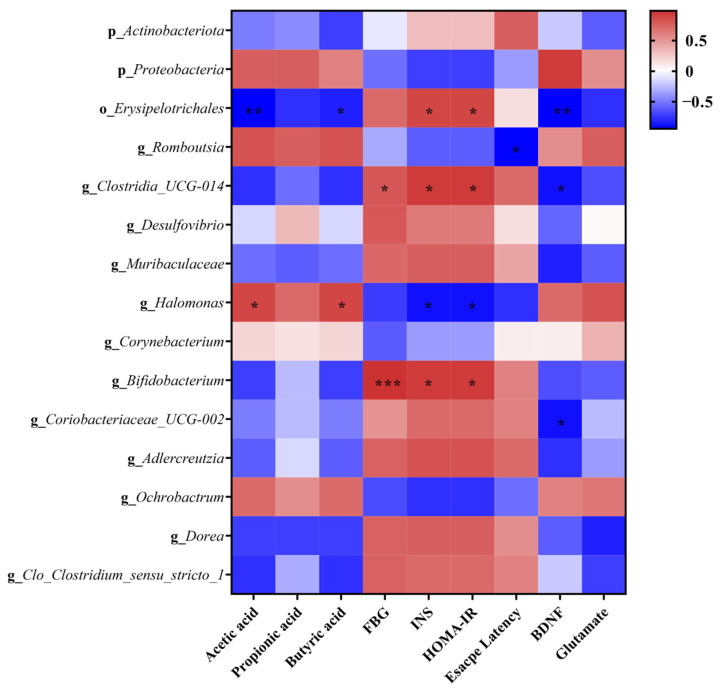
Spearman correlation analysis. Data are presented as the mean ± SD. * *p* < 0.05, ** *p* < 0.01, *** *p* < 0.001.

Spearman correlation analysis was conducted to demonstrate the association between the representative gut microbiota, microbial metabolites, and biochemical parameters. As shown in Figure 7, *Romboutsia*, *Halomonas*, *Corynebacterium*, *Helicobactor*, and *Ochrobactrum* exhibited a negative correlation with the escape latency of rats, fasting blood glucose, fasting insulin in serum, and HOMA-IR. Conversely, these bacterial genera demonstrated a positive correlation with fecal SCFA content, as well as serum BDNF and glutamate levels. However, contrasting trends were observed for *Clostridia_UCG-014*, *Desulfovibrio*, *Muribaculaceae*, *Bifidobacterium*, *Coriobacteriaceae_UCG-002*, *Adlercreutzia*, *Dorea*, and *Clo_Clostridium_sensu_stricto_1*.

Since LM+HT enhanced butyric acid levels in the feces of DM rats, the mechanism of sodium butyrate (NaB) was further confirmed in MG-induced PC-12 cells in vitro. Firstly, the cytotoxicity of NaB on PC-12 cells was detected by a CCK-8 assay (Figure S8A). Concentrations below 2.5 mM showed no cytotoxic effects on PC-12 cells. The cytotoxicity of methylglyoxal (MG) was detected by an CCK-8 assay, as shown in Figure S8B. The concentration of MG used for inducing damage in PC-12 cells was optimized to 0.5 mM for 48 h, as referred to in our previous study [26], in subsequent experiments. The protection of NaB in MG-induced PC-12 cells was detected by a CCK-8 assay. Treatment with NaB at concentrations ranging from 30 to 150 μM significantly attenuated the cytotoxicity induced by MG (Figure S8B) (*p* < 0.05). Therefore, a concentration of 150 μM NaB was selected for subsequent mechanistic studies.

**Figure 8:**
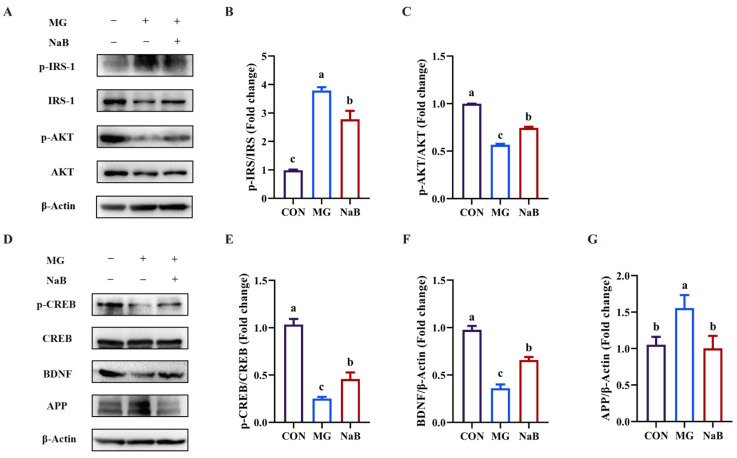
Effect of butyrate on methylglyoxal-induced PC-12 cells in vitro. Western blotting (**A**) and quantitative analysis of p-IRS-1/IRS-1 (**B**) and p-AKT/AKT (**C**) in PC-12 cells. Western blotting (**D**) and quantitative analysis of p-CREB/CREB (**E**), BDNF (**F**), and APP (**G**) in PC-12 cells. Data are presented as the mean ± SD. Groups sharing the same letter are not significantly different from each other, whereas groups with different letters exhibit statistically significant differences (*p* < 0.05). CON, control group. MG, 1 mM methylglyoxal group. NaB, 150 μM sodium butyrate group.

As shown in Figure 8, the protein expression levels of IRS and p-AKT were decreased in PC-12 cells, while p-IRS markedly increased in the MG group compared to the CON group (*p* < 0.05). Treatment with NaB significantly upregulated the expression of p-AKT and IRS while downregulating the expression of p-IRS in MG-induced PC-12 cells (*p* < 0.05). Moreover, treatment with MG inhibited the expression of p-CREB and BDNF in PC-12 cells (*p* < 0.05), which was reversed by NaB treatment (*p* < 0.05). Additionally, there was a significant increase in APP expression in the MG group compared to the CON group (*p* < 0.05). However, NaB treatment inhibited the expression of APP (*p* < 0.05).

## 3. Discussion

In recent years, numerous studies have reported an association between diabetes and cognitive decline, which poses a significant risk for AD and other forms of dementia [27,28,29,30]. However, the efficacy of metformin monotherapy as an excellent hypoglycemic drug for cognitive impairment in diabetes remains limited and sometimes controversial while also being accompanied by adverse effects. This phenomenon was also observed in the MWM test in this study, where the administration of 300 mg/kg metformin did not affect the escape latency of diabetic rats (Figure 1). Drug combinations are characterized by multi-target effects, enhanced drug efficacy, and reduced side effects, and they have been applied in interventions for diabetes and its complications. In the current study, the combination of hesperetin and a low dose of metformin (a third of the conventional dose of metformin) effectively reduced blood glucose levels and insulin resistance in DM rats. Furthermore, hesperetin enhanced the efficacy of metformin in ameliorating memory decline and the histological damage in the hippocampus caused by diabetes. To summarize, the combination of hesperetin and metformin demonstrated promising potential as a therapeutic strategy for alleviating cognitive impairment associated with diabetes. Furthermore, this combination enhanced efficacy while mitigating the adverse effects associated with monotherapy.

It is well recognized that insulin resistance is the leading feature of T2DM [31]. Recent research has provided substantial evidence linking insulin resistance to cognitive decline [32]. AKT, a crucial molecule involved in neuroprotection mediated by insulin signaling, has been reported to activate transcription factors such as CREB [33]. The expression of CREB was highly abundant in the hippocampus region of the brain, and phosphorylated CREB upregulated the transcription of BDNF downstream [34], which is essential for memory formation. In this work, the combination of hesperetin and metformin repaired the insulin signaling pathway and activated CREB downstream, leading to increased transcriptional and protein expression of BDNF. These findings provided novel insights into the potential mechanism underlying the alleviation of cognitive impairment through the supplementation of hesperetin with metformin in diabetic rats.

The aggregation of β-amyloid protein (Aβ) in the brain is a pivotal pathological factor of AD [35]. BACE-1 is an essential enzyme responsible for the proteolytic processing of APP, leading to the generation of Aβ peptides [36]. The downregulation of BACE-1 has been widely recognized as an effective therapeutic means for AD [37]. It has been found that insulin resistance increased APP metabolism and BACE-1 expression [38]. However, metformin, which is known to increase insulin resistance, activated the transcription of both BACE-1 and APP, potentially explaining its unsatisfactory therapeutic effect on the cognitive impairment when used alone [39]. The exact mechanism behind this phenomenon remains unclear. Similar results were also observed in our in vivo study, where the expression of BACE-1 was significantly higher in the metformin group with the conventional dosage than in both the DM group and LM group (Figure 4I–K). An in vitro study reported a reduction in the expression of BACE-1 and APP in advanced glycation end products (AGE)-induced SH-SY5Y cells due to hesperetin [40], but no in vivo results were documented. In our study, the supplementation of hesperetin with metformin significantly reduced the expression of BACE-1 and APP in vivo, suggesting that the combination improved central insulin resistance and potentially reduced the metformin dosage as a mechanism for enhancing metformin’s efficacy in improving diabetes-induced cognitive impairment. This further highlights the potential of the drug combination in mitigating the adverse effects associated with monotherapy.

The regulation of the gut microbiota has long been recognized as an effective approach to mitigating the development of diabetes [41]. A growing body of evidence consistently supports the existence of a microbiota–gut–brain axis, which establishes bidirectional communication between the gut and brain, exerting influence on cognitive function through the modulation of the gut microbiota [42]. Previous studies have documented the association between the regulation of *Romboustia* and *Bifidobacterium* and cognitive impairment. For example, a study conducted on elderly rural females in northwest China observed a decrease in the abundance of *Romboutsia* at the genus level among individuals with impaired cognition. Furthermore, although *Bifidobacterium* is widely considered a beneficial bacterium [43], it exhibited a positive correlation with cognitive impairment induced by exposure to pollutants [44]. A similar tendency was also observed in the finding that procyanidin improved cognitive impairment in aging mice by inhibiting the abundance of *Bifidobacterium* [45]. In our results, at the genus level, the abundance of *Bifidobacterium* increased, while that of *Romboutsia* decreased in the DM group. Notably, these trends were successfully reversed in the LM+HT group, where the abundance of *Bifidobacterium* decreased and that of *Romboutsia* increased (Figure 5). These results confirmed the opposing role of Bifidobacterium in cognitive impairment and suggested that the abundance of *Romboutsia* and *Bifidobacterium* at the genus level could serve as biomarkers for the early screening of cognitive impairment. Furthermore, it was noted that there was an increment in the *Proteobacteria* phylum in the CM group, though it decreased significantly in the LM+HT group (Figure 6A). A previous study indicated that this pathogenic bacterium was closely associated with gastrointestinal reactions to metformin [46,47]. Our results suggested that hesperetin supplementation might reduce the side effects of metformin. Thus, the results obtained in the gut microbiota analysis revealed that the modulation of gut microbiome changes might underline the mechanism through which the combination of hesperetin and metformin improved cognitive impairment and reduced side effects compared with metformin alone. Additionally, fecal microbiota transplantation will be imperative in the future to validate the pivotal role of gut microbiota regulated by this intervention in alleviating diabetic cognitive impairment.

**Figure 9:**
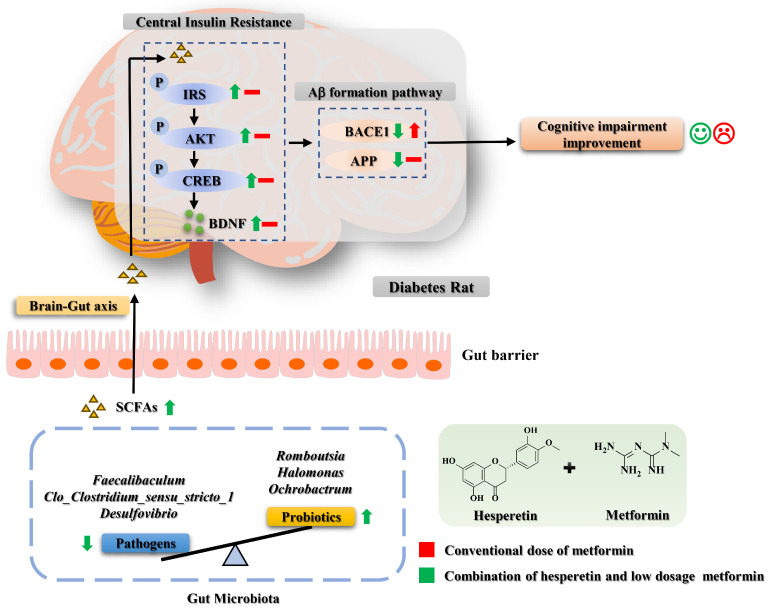
Proposed mechanism of hesperetin supplementation with metformin against cognitive impairment in T2DM.

SCFAs are crucial metabolites produced by the gut microbiota. Acetate, propionate, and butyrate collectively account for 80% of all known SCFAs and are closely related to the amelioration of insulin resistance [48]. Valeric acid is also a kind of SCFA and is produced in minor quantities during the fermentation of dietary fiber [49]. Recently, SCFAs have been identified as pivotal components mediating the microbiota–gut–brain axis [50]. For instance, valeric acid showed a positive effect on ameliorating dementia [51], and β-hydroxybutyrate and sodium butyrate were reported to alleviate oxidative stress in AD rats or cognitive impairment in AD mice by acting on astrocytes [52,53]. *Romboutsia* has been reported to be involved in butyrate production [54]. However, whether the upregulation of the level of SCFAs was related to the improvement of cognitive impairment in DM mice remains unexplored. In our study, in vivo experiments demonstrated that the supplementation of hesperetin with metformin elevated acetic acid, propionic acid, and butyric acid content, leading to improved cognitive impairment in DM rats through the protection of insulin signaling and the activation of CREB/BDNF (Figure 6). These results were further confirmed by an in vitro study, where treatment with butyrate was observed to enhance cell viability in MG-induced PC-12 cells (Figure 8). Thus, the regulation of insulin signaling and CREB/BDNF pathways in the hippocampus via SCFAs, particularly butyrate, was proposed as the mechanism by which the combination of hesperetin and metformin improved cognitive impairment in DM rats through the gut–brain–microbiota axis (Figure 9). However, the role of butyrate in diabetic cognitive impairment in vivo remains to be explored.

This study primarily provided promising insights into a combination therapy with hesperetin and metformin for diabetes-associated cognitive impairment, focusing on animal models. However, it is still necessary to validate the efficacy of this combination in human clinical trials. Furthermore, it was reported that 12 weeks of daily supplementation with 500 mg of citrus extract, which is high in flavonoids, including hesperetin, had a positive effect on intestinal metabolic responses in human volunteers without obvious side effects [55], suggesting the safety of hesperetin. Nevertheless, the potential long-term effects and safety profile of the combination of hesperetin and metformin will still need to be investigated.

## 4. Materials and Methods

### 4.1. Chemicals and Reagents

Hesperetin (purity ≥ 98%) was obtained from Chengdu Kanghui Bio-Technology Co., Ltd. (Chengdu, China). Metformin was obtained from Beijing Solarbio Science and Technology Co., Ltd. (Beijing, China). STZ was purchased from MedChemExpress (Monmouth Junction, NJ, USA).

### 4.2. Animals and Experimental Treatments

**Figure 10:**
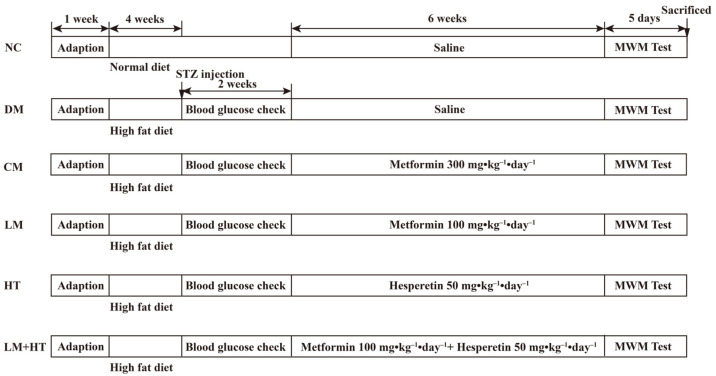
Experimental design.

Four-week-old male Sprague Dawley (SD) rats were obtained from Liaoning Changsheng Biotechnology Co., Ltd. (Benxi, China). Rats were housed in a 12 h light and dark cycle at a temperature of 25 ± 1 °C and a relative humidity of approximately 55 ± 5% and with free access to tap water and food. After acclimatization with an unrestricted diet for one week, the rats were randomly separated into six groups: normal diet control (NC, an equal volume of saline), diabetes mellitus (DM, an equal volume of saline), conventional-dose metformin (CM, 300 mg·kg^−1^·day^−1^), low-dose metformin (LM, 100 mg·kg^−1^·day^−1^), hesperetin (HT, 50 mg·kg^−1^·day^−1^), and low-dose metformin+hesperetin (LM+HT, 100 mg·kg^−1^·day^−1^ metformin+50 mg·kg^−1^·day^−1^ hesperetin) groups. The NC group was fed with a standard diet (*n* = 8), and the other five groups were fed with a high-fat diet (45% energy from fat, D12451). Diets were purchased from SYSE Biotechnology Co., Ltd. (Changzhou, China). Rats fed with high-fat diet after four weeks were injected with a single dose of STZ (30 mg/kg) intraperitoneally (i.p.) dissolved in 0.1 M citrate buffer (pH = 4.5) following overnight fasting. Rats with the blood glucose taken from the tail vein over 16.7 mmol/L at 7 and 14 days post-injection were considered diabetic (*n* = 8 in each group). Body weight was recorded weekly throughout the experiment. Following a six-week intervention, the Morris water maze test was subsequently conducted. Rats were fasted overnight and euthanized through CO_2_ exposure after the behavior analysis, and then the serum and tissues were collected and either flash-frozen in liquid nitrogen and stored at −80 °C for further analysis or stored in 4% paraformaldehyde for histological analysis. The experimental design is shown in Figure 10.

### 4.3. Morris Water Maze (MWM)

The MWM has been widely used to evaluate rats’ memory and spatial learning. The rats were trained in a circular pool with a diameter of 150 cm and a height of 50 cm, containing an escape platform that was 10 cm in diameter and 1 cm deep. All the rats were trained with four trials per day for 4 consecutive days. The escape latency, that is, the time to find the platform, was recorded. The rats that failed to find the hidden platform within 60 s were allowed to stay there for a further 5 s, and the escape latency was recorded as 60 s. On the 5th day, a single 60 s probe trial was performed after removing the platform, and the escape latency, the number of rats crossing the previous platform position, and the time spent in the target quadrant were recorded and analyzed.

### 4.4. Oral Glucose Tolerance Tests (OGTTs)

An oral glucose tolerance test was performed by gavage of glucose (2 g·kg^−1^) to 12 h-fasted rats. Blood glucose taken from the tail vein was determined with a glucometer (Roche ACCU-CHEK, Basel, Switzerland) 0, 30, 60, 90 and 120 min after glucose administration. The area under the curve (AUC) was calculated as shown in the equation below:

(1) $$AUC=(FBG+2\times {PBG}_{30min}+2\times {PBG}_{60min}+2\times {PBG}_{90min}+{PBG}_{120min})\times 30\;min\times 0.5$$

### 4.5. Insulin Tolerance Tests (ITTs)

Insulin (1 IU·kg^−1^) was injected intraperitoneally to 6h-fasted rats for the insulin tolerance test. Blood glucose taken from the tail vein was determined by a glucometer (Roche ACCU-CHEK) at times 0, 30, 60, 90 and 120 min after insulin rejection. The AUC of glucose was calculated as Equation (1).

### 4.6. Histopathological Analysis

After the behavioral test, the rats were anesthetized and sacrificed. The brains were collected and fixed in 4% paraformaldehyde for 24 h. Then, the brains were embedded in paraffin and cut into 4 μm sections. Next, the sections were deparaffinized and stained with hematoxylin–eosin (H&E) and nissl and were observed under a microscope (OLYMPUS IX83, Tokyo, Japan).

### 4.7. Determination of Serum Indicators

Serum was used to determine glycosylated serum protein (GSP, Nanjing Jiancheng Bioengineering Institute, Nanjing, China), glutamate (Nanjing Jiancheng Bioengineering Institute), and BDNF (Elabscience, Wuhan, China) following the instructions.

### 4.8. Determination of Insulin In Vivo

Blood samples were collected and centrifuged 3000 rpm, and supernatants were stored at −80 °C. The levels of insulin in serum were determined using the ELISA kit (Elabscience, Wuhan, China) following the instructions. The homeostasis model assessment of insulin resistance (HOMA-IR) was calculated according to the following equation:

(2) HOMA−IR=fasting insulin (mIU/L)×fasting blood glucose (mmol/L)/22.5

### 4.9. Quantitative Real-Time PCR (qRT-PCR)

The total RNA of hippocampus tissues was extracted and reverse-transcribed using RNAiso Plus and one-step RT-PCR kits, following the manufacturer’s instructions (Takara, Tokyo, Japan). *Bdnf* was assayed by quantitative real-time PCR. Primers were designed by Shanghai Sangon Biotech and are presented in Supplementary Table S1. The amplification conditions were as follows: 95 °C for 30 s, followed by 40 cycles of 95 °C for 5 s and 58 °C for 34 s. The relative expression level of target genes was normalized to the expression of *β-actin* and analyzed by the 2^−ΔΔCt^ method.

### 4.10. Western Blot

RIPA lysis buffer containing the phosphatase inhibitor cocktail and ethylenediaminetetraacetic acid-free protease was utilized for the lysis of cells or brain tissues and then centrifuged at 12,000 rpm for 10 min. The supernatant was collected and boiled in loading buffer. The BCA protein quantitation kit was used to measure the concentration of protein. In brief, protein samples at the same concentration were separated by sodium dodecyl sulfate–polyacrylamide gel electrophoresis (SDS-PAGE) and then transferred to polyvinylidene fluoride (PVDF) membranes. Next, the membranes were blocked with 5% bovine serum albumin (BSA) solution for 2 h at room temperature and then incubated with primary antibodies at 4 °C overnight. After this, the membranes were further incubated with horseradish peroxidase-conjugated secondary antibodies for 1 h at room temperature. Ultimately, the immunoreactive bands were developed with ECL detection reagents and quantified using Image J 1.53 software, and the results were normalized. The primary antibodies used in this experiment included IRS-1 (1:1000, Abclonal, Wuhan, China, A0245), p-IRS-1 (1:1000, CST, Boston, MA, USA, 2381), PI3K (1:1000, WanleiBio, Shenyang, China, WL02240), AKT (1:1000, Abclonal, A22770), p-AKT (1:1000, CST, 4060), CREB (1:1000, Abclonal, A10826), p-CREB (1:1000, Abclonal, AP0019), BDNF (1:1000, Abclonal, A16299), APP (1:1000, Abclonal, A17911), BACE-1 (1:1000, Beyotime, Shanghai, China, AF6273), and β-actin (1:1000, Proteintech, Wuhan, China, 66009-1-Ig).

### 4.11. 16S rRNA Sequencing

The fecal samples were collected and handed over to Hangzhou Guhe Information Technology Co., Ltd. (Hangzhou, China), for 16S rRNA gene sequencing. The total DNA was extracted from fecal samples, and the V4 hypervariable region of the 16S rRNA was amplified (515F: GTGCCAGCMGCCGCGGTAA and 806R: GGACTACHVGGGTWTCTAAT). The libraries that passed the test were then subjected to high-throughput sequencing using the Illumina NovaSeq platform (Illumina, San Diego, CA, USA). The QIIME2 training classifier method was used to analyze the sequences.

### 4.12. Transcriptome Analysis

Transcriptome analysis was performed at LC-Bio Technology Co., Ltd. (Hanzhou, China) The total RNA of hippocampus tissue was extracted using RNAiso plus (Takara) reagent. The quality of the RNA was evaluated by a NanoDrop 2000 (Thermo Fisher Scientific, Waltham, MA, USA) and Agilent Bioanalyzer 2100 system (Santa Clara, CA, USA). Then, 1 μg of RNA was used to construct the library with the Illumina Novaseq 6000 platform. Bioinformatic analysis was performed using the OmicStudio tools at https://www.omicstudio.cn/tool (accessed on 30 January 2024).

### 4.13. Quantification of SCFAs

Short-chain fatty acids (SCFAs) in fecal samples were determined by gas chromatography (GC), as previously described [56]. The sample was thawed on ice, and about 50 mg of the sample was added to 1 mL of anhydrous methanol. The supernatant was filtered through a 0.22 μm filter (Millipore, Birica, MA, USA), stored in 2 mL screw cap vials, and analyzed on the Agilent gas chromatography system.

### 4.14. Cell Culture

Rat pheochromocytoma PC-12 cells were obtained from the Chinese Academy of Sciences Cell Bank (Shanghai, China) and cultured in RPMI 1640 (Gibco, Thermo Fisher Scientific, Waltham, MA, USA) supplemented with 10% fetal bovine serum (FBS) and 100 U/mL penicillin and 100 g/mL streptomycin in a humidified 5% CO_2_ atmosphere at 37 °C.

### 4.15. Cell Counting Kit-8 (CCK-8) Assay

Cell viability was detected by a CCK-8 assay. PC-12 cells were seeded into 96-well plates (1 × 10^4^ cells/well) overnight and were treated with sodium butyrate (NaB) alone or co-cultured with methylglyoxal (MG). At the end of the incubation for 24 h, 10 μL of CCK-8 reagent (APE×bio, Houston, TX, USA) was added into each well, and cells were incubated for another 1 h at 37 °C. The absorbance at 450 nm was measured by a microplate reader (SpectraMax M2e, Molecular Device, San Jose, CA, USA).

### 4.16. Statistical Analysis

All bar plots in this study were generated with GraphPad Prism 8.3 (GraphPad Software, La Jolla, CA, USA). An analysis of differential significance was performed by SPSS 26.0 software (IBM SPSS, Chicago, IL, USA). The significant difference was determined by one-way analysis of variance (ANOVA) with criteria as *p* value < 0.05 [57,58]. The Tukey test was used for subsequent analysis.

## 5. Conclusions

Our study demonstrated that combination of hesperetin and metformin effectively attenuated hyperglycemia and cognitive impairment associated with diabetes. Furthermore, this supplementation regimen regulated gut microbiota composition and SCFAs levels in vivo, thereby modulating the insulin signaling and CREB/BDNF pathways in the hippocampus. The findings of this study provide valuable insights into the potential effects of hesperetin used in supplementary treatment in addressing cognitive impairment induced by type 2 diabetes via the gut–brain axis.

## Data Availability

The data that support the findings of this study are available from the corresponding author upon reasonable request.

## Supplementary Materials

The following supporting information can be downloaded at https://www.mdpi.com/article/10.3390/ijms26051923/s1.

## Abbreviations

The following abbreviations are used in this manuscript:

STZ	Streptozotocin
T2DM	Type 2 diabetes mellitus
SCFAs	Short-chain fatty acids
CREB	Cyclic-AMP response element-binding protein
BDNF	Brain-derived neurotrophic factor
BACE1	Beta-site amyloid precursor protein cleaving enzyme 1
AD	Alzheimer’s disease
MWM	Morris water maze
OGTT	Oral glucose tolerance tests
AUC	Area under the curve
FBG	Fasting blood glucose
PBG	Postprandial blood glucose
ITT	Insulin tolerance tests
H&E	Hematoxylin–eosin
GSP	Glycosylated serum protein
HOMA-IR	Homeostasis model assessment of insulin resistance
qRT-PCR	Quantitative real-time PCR
SDS-PAGE	Sodium dodecyl sulfate-polyacrylamide gel electrophoresis
BCA	Bicinchoninic acid
PVDF	Polyvinylidene fluoride
BSA	Bovine serum albumin
IRS	Insulin receptor substrates
PI3K	Phosphatidylinositol 3-kinase
AKT	Protein kinase B
APP	Amyloid precursor protein
PCR	Polymerase chain reaction
GC	Gas chromatography
CON	Control group
MG	Methylglyoxal
NaB	Sodium butyrate
NC	Normal diet control
DM	Diabetes mellitus
CM	Conventional-dose metformin
LM	Low dose metformin
HT	Hesperetin
LM+HT	Low dose metformin+hesperetin
CCK-8	Cell counting kit-8
DG	Dentate gyrus
GSEA	Gene set enrichment analysis
KEGG	Kyoto encyclopedia of genes and genomes
FINS	Fasting insulin
