# Supplementary material for: Hesperetin-Enhanced Metformin to Alleviate Cognitive Impairment via Gut–Brain Axis in Type 2 Diabetes Rats
Source: Int J Mol Sci. 2025 Feb 23;26(5):1923. doi: 10.3390/ijms26051923 (PMC11900253; doi:10.3390/ijms26051923)
Supplement: Supplementary file 1 [file ijms-26-01923-s001.zip › ijms-3416811-supplementary.pdf]

Supporting Information

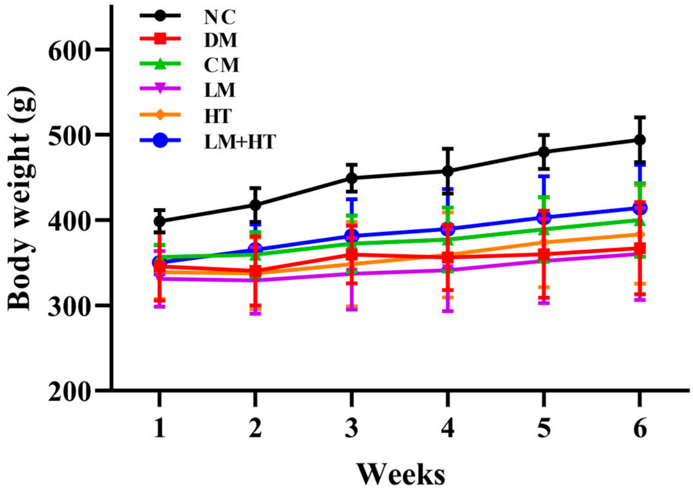

Figure S1 Effect of combination of hesperetin and metformin on body weight in diabetes rats. NC, normal diet control group. DM, diabetes mellitus group. CM, conventional dose metformin (300 mg•kg^{-1}•day^{-1}) group. LM, low-dose metformin (100 mg•kg^{-1}•day^{-1}) group. HT, hesperetin (50 mg•kg^{-1}•day^{-1}) group. LM+HT, low-dose metformin+hesperetin (100 mg•kg^{-1}•day^{-1} metformin+50 mg•kg^{-1}•day^{-1} hesperetin).

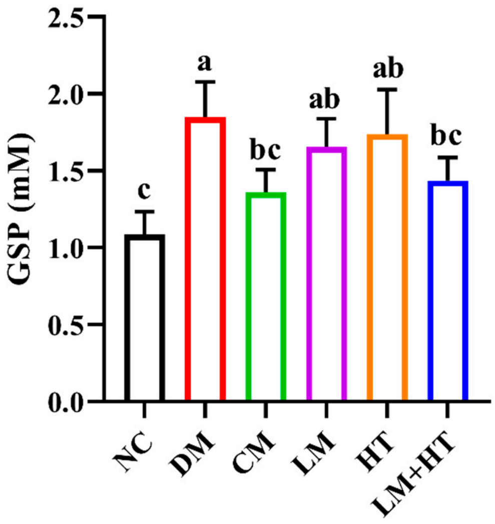

Figure S2 Effect of combination of hesperetin and metformin on glycosylated serum protein (GSP) in serum. Data were presented as the mean ± SD. Different letters represented significance between the two groups (*p*<0.05). NC, normal diet control group. DM, diabetes mellitus group. CM, conventional dose metformin (300 mg•kg^{-1}•day^{-1}) group. LM, low-dose metformin (100 mg•kg^{-1}•day^{-1}) group. HT, hesperetin (50 mg•kg^{-1}•day^{-1}) group. LM+HT, low-dose metformin+hesperetin (100 mg•kg^{-1}•day^{-1} metformin+50 mg•kg^{-1}•day^{-1} hesperetin).

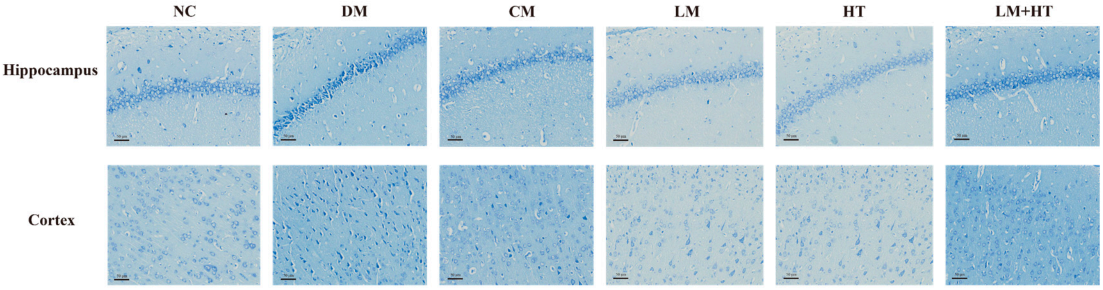

Figure S3 Effect of combination of hesperetin and metformin on nissl staining of hippocampus. NC, normal diet control group. DM, diabetes mellitus group. CM, conventional dose metformin (300 mg•kg^{-1}•day^{-1}) group. LM, low-dose metformin (100 mg•kg^{-1}•day^{-1}) group. HT, hesperetin (50 mg•kg^{-1}•day^{-1}) group. LM+HT, low-dose metformin+hesperetin (100 mg•kg^{-1}•day^{-1} metformin+50 mg•kg^{-1}•day^{-1} hesperetin).

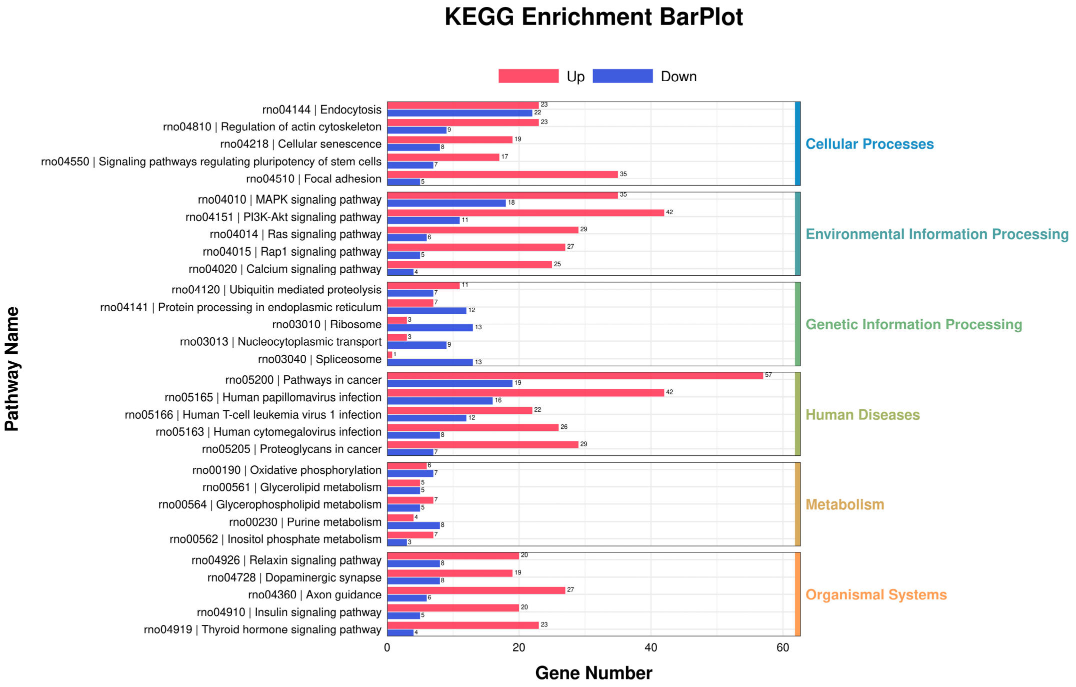

Figure S4 Kyoto Encyclopedia of Genes and Genomes (KEGG) enrichment of transcriptome analysis.

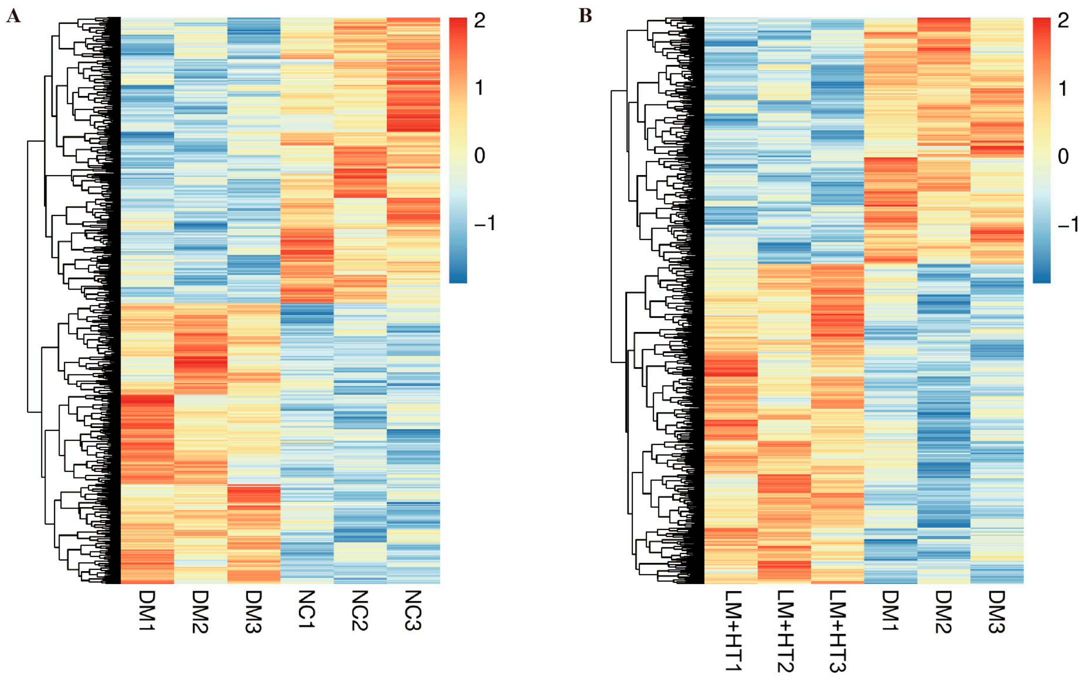

Figure S5 Heatmap of differentially expressed genes in transcriptome analysis. (A) DM vs NC. (B) LM+HT vs DM. NC, normal diet control group. DM, diabetes mellitus group. LM+HT, low-dose metformin+hesperetin (100 mg•kg^{-1}•day^{-1} metformin+50 mg•kg^{-1}•day^{-1} hesperetin).

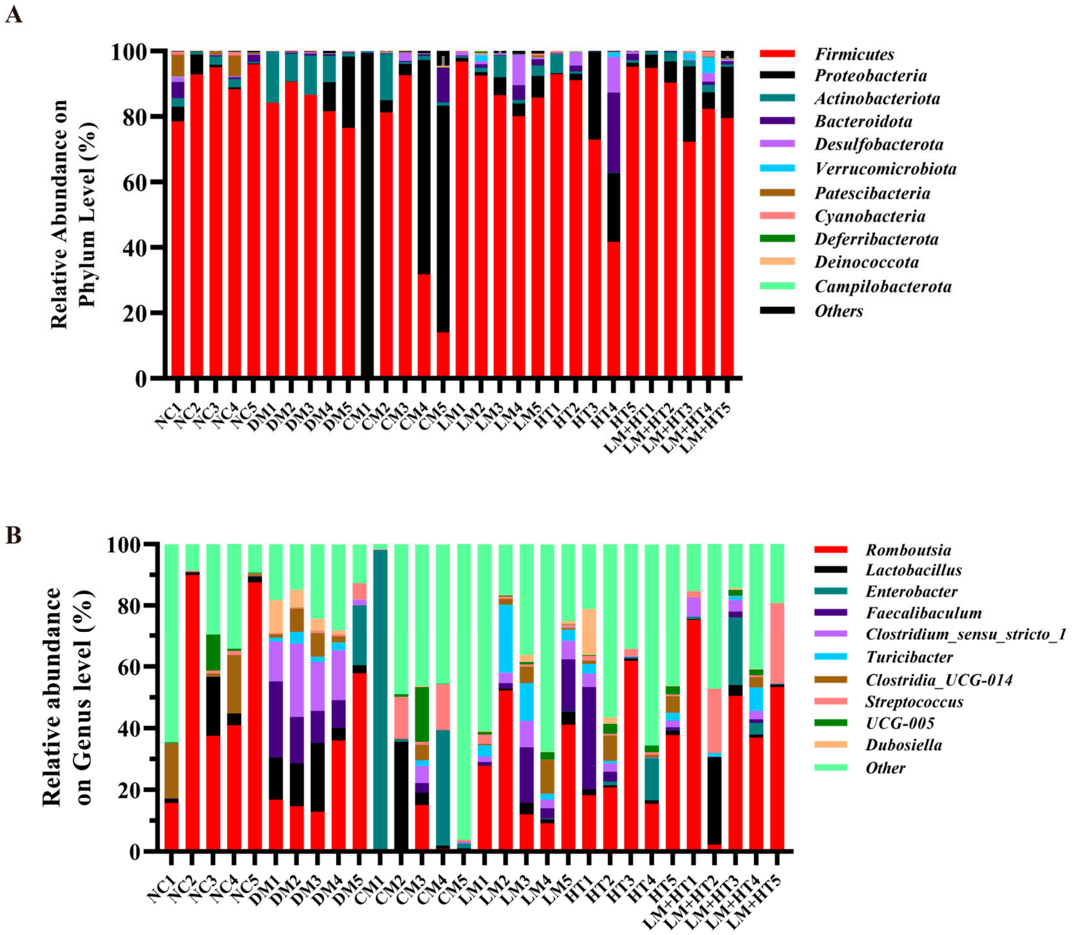

Figure S6 Relative abundance in Phylum level (A) and Genus level (B). NC, normal diet control group. DM, diabetes mellitus group. CM, conventional dose metformin (300 mg•kg^{-1}•day^{-1}) group. LM, low-dose metformin (100 mg•kg^{-1}•day^{-1}) group. HT, hesperetin (50 mg•kg^{-1}•day^{-1}) group. LM+HT, low-dose metformin+hesperetin (100 mg•kg^{-1}•day^{-1} metformin+50 mg•kg^{-1}•day^{-1} hesperetin).

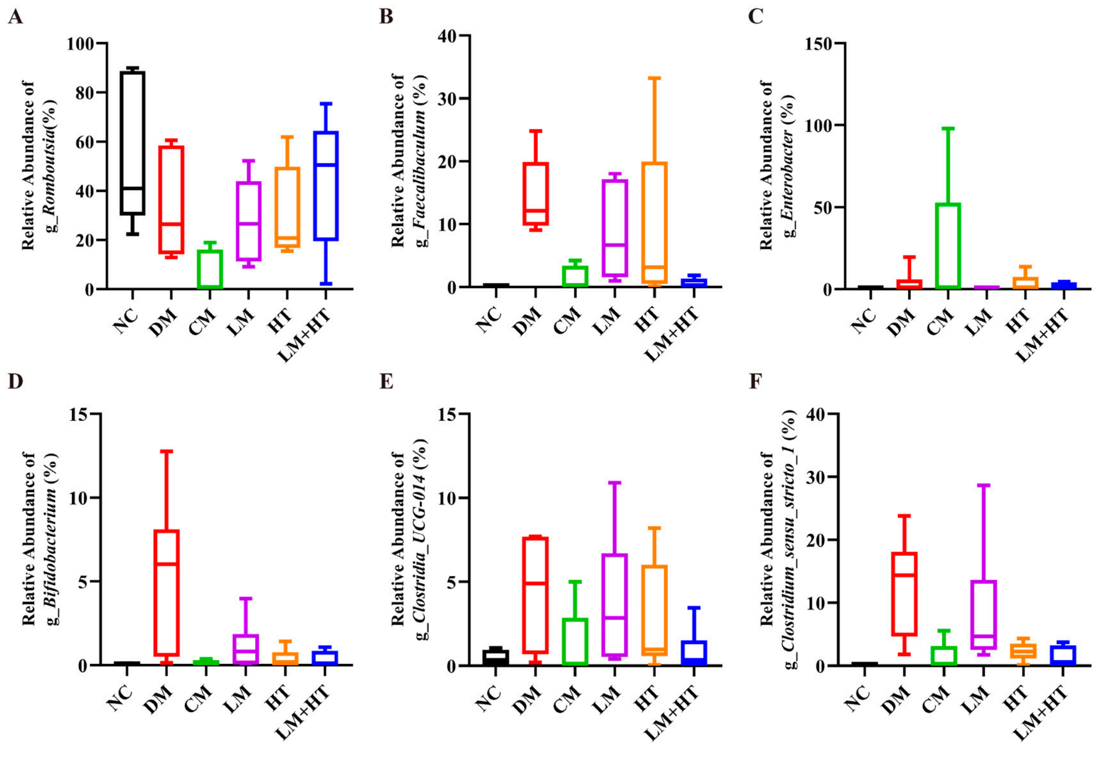

Figure S7 Effect of combination of hesperetin and metformin on relative abundance of (A) g_*Romboutsia*, (B) g_*Faecalibaculum*, (C) g_*Enterobacter*, (D) g_*Bifidobacterium*, (E) g_*Clostridia_UCG-014*, (F) g_*Clo_Clostridium_sensu_stricto_1*. NC, normal diet control group. DM, diabetes mellitus group. CM, conventional dose metformin (300 mg•kg^{-1}•day^{-1}) group. LM, low-dose metformin (100 mg•kg^{-1}•day^{-1}) group. HT, hesperetin (50 mg•kg^{-1}•day^{-1}) group. LM+HT, low-dose metformin+hesperetin (100 mg•kg^{-1}•day^{-1} metformin+50 mg•kg^{-1}•day^{-1} hesperetin).

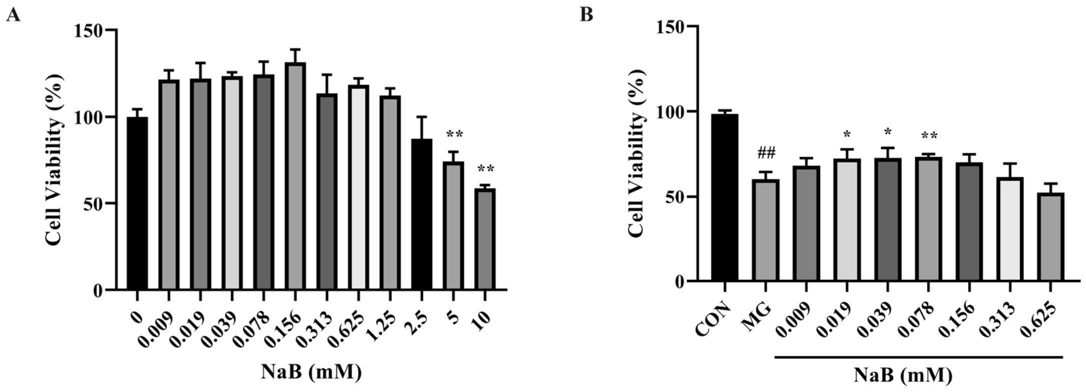

Figure S8 Cell viability. (A) Cell viability of treatment with NaB in PC-12 cells. Data were presented as the mean ± SD. ***p*<0.001 vs 0 group. (B) Cell viability of treatment with MG and NaB in PC-12 cells. Data were presented as the mean ± SD. ##*p*<0.01 vs CON group. **p*<0.05 vs MG group. **p*<0.01 vs MG group. CON, control group. MG, methylglyoxal group. NaB, sodium butyrate group.

Table S1 Primer sequences used in the quantitative real-time PCR analysis

**Target mRNA**	**Primer sequence (5'-3')**	**Fragment size (bp)**
Bdnf	F: TGGAACTCGCAATGCCGAACTAC<br>R: TCCTTATGAACCGCCAGCCAATTC	88
β-Actin	F: TGTCACCAACTGGGACGATA<br>R: GGGGTGTTGAAGGTCTCAAA	165
